# Bio-Intensive Tactics for the Management of Invasive Fall Armyworm for Organic Maize Production

**DOI:** 10.3390/plants12030685

**Published:** 2023-02-03

**Authors:** Manikyanahalli Chandrashekara Keerthi, Sachin Suresh Suroshe, Sagar Doddachowdappa, Kadanakuppe Thammayya Shivakumara, Hosapura Shekhararaju Mahesha, Virendra Singh Rana, Ankita Gupta, Ajith Murukesan, Ryan Casini, Hosam O. Elansary, Najam Akhtar Shakil

**Affiliations:** 1 Division of Entomology, ICAR-Indian Agricultural Research Institute, Pusa, New Delhi 110012, India; 2 Seed Technology Division, ICAR-Indian Grassland and Fodder Research Institute, Jhansi 284003, India; 3 Division of Crop Protection, ICAR-Indian Institute of Horticultural Research, Bengaluru 560089, India; 4 ICAR–National Bureau of Agricultural Insect Resources, Bengaluru 560024, India; 5 Division of Agricultural Chemicals, ICAR-Indian Agricultural Research Institute, Pusa, New Delhi 110012, India; 6 School of Public Health, University of California, Berkeley, 2121 Berkeley Way, Berkeley, CA 94704, USA; 7 Department of Plant Production, College of Food & Agriculture Sciences, King Saud University, P.O. Box 2460, Riyadh 11451, Saudi Arabia

**Keywords:** biocontrol, *Chelonus formosanus*, botanicals, fall armyworm, intercropping, *Telenomus remus*

## Abstract

*Spodoptera frugiperda* (J.E. Smith) (Lepidoptera: Noctuidae) is an invasive pest native to the American continent. The present study focused on bio-intensive tactics like intercropping, using natural enemies, botanical insecticides and biopesticides for managing *S. frugiperda* for the organic production of maize in Indian conditions. A total of eight different parasitoids attacking the different stages of *S. frugiperda viz.,* eggs and larvae were found in the study area. The total parasitism rate due to all the parasitoids ranged from 28.37 to 42.44%. The egg-larval parasitoid, *Chelonus formosanus* Sonan (Hymenoptera: Braconidae) was the dominant parasitoid (12.55%), followed by *Chelonus* nr. *blackburni* (Hymenoptera: Braconidae) (10.98%) and *Coccygydium* sp. (4.85%). About 36.58 percent of the egg masses collected was parasitized by egg parasitoids, among which *Telenomus remus* (Nixon) (Hymenoptera: Scelionidae) was the dominant parasitoid. The botanicals insecticides such as citronella and annona extract were most effective, resulting in 100% mortality of FAW larvae (168 h after treatment). The essential oil of garlic (100%) was found highly effective in inhibiting egg hatching, followed by geraniol (90.76%). The maize intercropped with lady’s finger (okra) recorded significantly the lowest pest infestation and recorded higher grain yield (6.17 q/ha) than other intercropping systems and control (5.10 q/ha). The overall bioefficacy of commercial biopesticides against the larvae of *S. frugiperda* was in the following order azadirachtin > *Metarhizium anisopliae* (Metch.) Sorokin (Hypocreales: Clavicipitaceae) > *Beauveria bassiana* (Balsamo) Vuillemin (Hypocreales: Clavicipitaceae) at 168 h after treatment.

## 1. Introduction

Fall Armyworm (FAW), *Spodoptera frugiperda* (J.E. Smith) (Lepidoptera: Noctuidae), is a highly polyphagous invasive pest native to the Americas, that has expanded its distribution from western to eastern hemisphere [1,2]. It was first discovered in Africa in 2016 and is now found in over 70 countries throughout Asia and Oceania [1,3,4]. Since its introduction, FAW has emerged as a serious threat to cereal crop productivity, particularly maize and sorghum, two of the major staple food crops of Asia and Africa’s smallholder farmers, threatening regional food security [5,6]. To contain the FAW spread, many African and Asian countries have recommended, distributed, and applied synthetic pesticides [7,8]. For instance, in 2017, Zimbabwe distributed nearly 102,000 L of pesticide worth $1.97 million USD to farmers [9]. Despite government subsidies, the use of synthetic insecticide as the sole control measure is unsustainable due to its high cost, increased pesticide resistance, pest resurgence, and risk to human health and the environment.

The use of cover crops/hedge rows/intercrops/flower strip crops in the main crop field to conserve beneficial insect fauna is known as habitat manipulation [10,11]. Conservation biological control is a method of customizing crop habitat to support and sustain the population of native parasitoids and predators for biological control of pests [12,13]. Intercrops not only help to reduce pest infestations but also improve soil fertility and serve as a refuge for parasitoids and predators [14,15]. Despite the availability of successful modules on the use of habitat manipulation for managing other maize borer pests such as stem borers [16], extensive research on agro-ecological approach for the management of Fall Armyworm under Indian conditions is lacking. Furthermore, using vegetable crops as intercrops to reduce the incidence is a novel concept as it also generates additional income for farmers [17].

The Fall Armyworm in India is attacked by various natural enemies [18], including insect parasitoids and predators, and entomopathogens such as fungi, bacteria, viruses, and nematodes. Efficient natural enemies of FAW could be identified and integrated into an Integrated Pest Management (IPM) system to achieve economic growth and environmental safety and sustainability paving the way for organic production [19]. Keerthi et al. [15] identified *Eocanthecona furcellata* (Wolff) (Hemiptera: Pentatomidae), as an efficient predator of FAW from central India and also, larval parasitoids like *Cotesia ruficrus* (Haliday) (Hymenoptera: Braconidae)*, Campoletis chlorideae* Uchida (Hymenoptera: Ichneumonidae)*,* and *Aleiodes* sp. from field [3]. *Chelonus formosanus* was identified as the most dominant egg-larval parasitoid of FAW in northern India by Sagar et al. [20]. Udayakumar et al. [21] and Navik et al. [22] also documented the distribution of egg parasitoids such as *Trichogramma chilonis* Ishii (Hymenoptera: Trichogrammatidae) and *Telenomus remus* (Nixon) (Hymenoptera: Scelionidae) in southern India. Sivakumar et al. [23] reported the natural occurrence of entomopathogens on FAW like *Bacillus thuringiensis* Berliner (Bacillales: Bacillaceae), *Nomuraea rileyi* (Farl.) Samson (Hypocreales: Clavicipitaceae), *Spfr*NPV (Lefavirales: Baculoviridae). Hence, we hypothesize that, identifying relatively dominant and efficient natural enemies for their use under field conditions might leads to development of bio-intensive management option for suppressing FAW in the India.

Effective control of the borer complex of maize in the America has mainly relied on the genetically modified maize hybrids expressing *Bt* insecticidal proteins for over a decade [24]. However, the management of FAW exclusively depends on the synthetic pesticides in Africa and Asia [8,25]. Because of high cost involved in pesticide control and risk to human health and the environment, the use of synthetic pesticides as the sole control measure is unsustainable in long run [26]. Botanicals are one of the suitable alternatives to synthetic pesticides and have a great potential to use under field conditions. Among botanicals, essential oils are complex secondary metabolite mixtures that evolved in plant defense mechanisms against insects and that, when extracted and applied exogenously, can confer insecticidal, repellent, or antifeedant activities [27]. Sombra et al. [28] evaluated the efficacy of three essential oils on the different stages of FAW, among which *Lippia origanoides* Kunth, registered an average of 97.8% ovicidal activity and 81.3% pupicidal activity. Negrini et al. [29] also documented 100% mortality of second instar larvae of FAW due to the application of *Corymbia citriodora* (Hook.) K.D. Hill & L.A.S. Johnson (Myrtales: Myrtaceae) and *Lippia microphylla* Cham. (Lamiales: Verbenaceae). Being natural in origin, a research on the phytosanitary use of botanicals derived from various aromatic plants has increased [30]. Owing to its revamped distribution range, it would be prudent to document the native biocontrol agents associated with the invasive pest and evaluate ecofriendly pest management options against the pest to develop an Integrated Pest Management (IPM) module in India.

## 2. Results

### 2.1. Field Parasitism by Egg and Larval Parasitoids of FAW

During the study period, a total of 287 egg masses and 2741 larvae were collected from maize fields during the main cropping season. Table 1 shows the total number of FAW larvae collected during each cropping season month, as well as the average parasitization rates. Six parasitoids emerged from field collected larvae, including two egg-larval parasitoids *viz*., *Chelonus formosanus* and *Chelonus* nr. *blackburni* (Hymenoptera: Braconidae) and four larval parasitoids *Coccygidium* sp. (Hymenoptera: Braconidae), *Temelucha* sp. (Hymenoptera: Ichneumonidae), *Cotesia ruficrus* (Haliday) (Hymenoptera: Braconidae) and *Campoletis chlorideae* Uchida (Hymenoptera: Ichneumonidae). The combined parasitism rate due to all the parasitoids ranged from 28.37 to 42.44, and the highest parasitism rate (42.44) was recorded during July, 2021.

**Figure 1:**
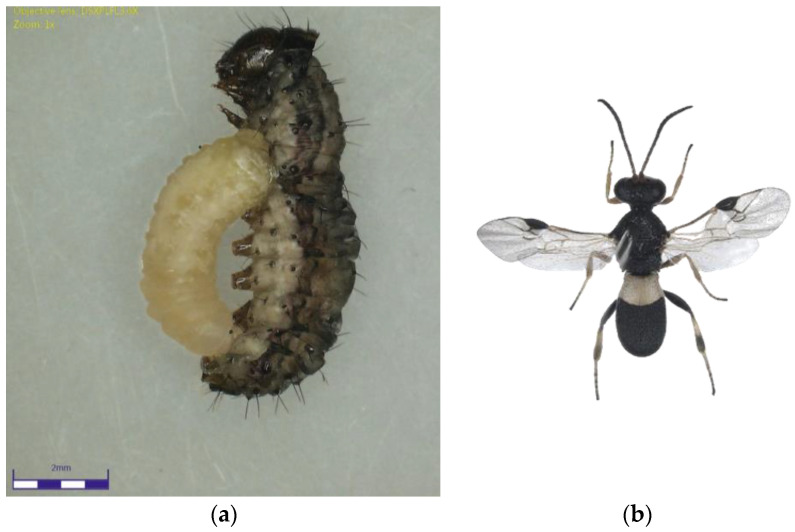
(**a**) *Chelonus* nr. *blackburni* larva emerging from the Fall Armyworm larvae; (**b**) *Chelonus* nr. *blackburni* adult.

**Figure 2:**
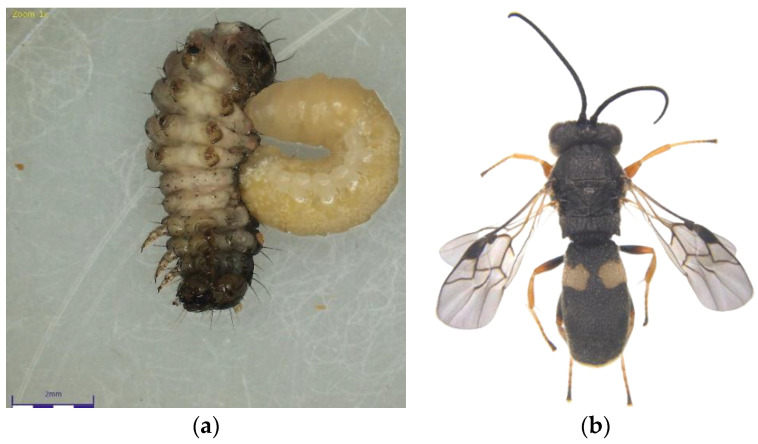
(**a**) *Chelonus formosanus* larva emerging from the Fall Armyworm larvae; (**b**) *C. formosanus* adult.

The *Chelonus* nr. *blackburni* was the most abundant and contributed the most to parasitism (18.57%) during July, 2021 (Figure 1a,b). During August and September, the most abundant parasitoid was *C. formosanus* (32.56 and 45.57%), which contributed significantly to the FAW’s natural mortality, respectively (Figure 2a,b). Even though *Coccygidium* sp. was active throughout the cropping season, it was most abundant (34.33%) in October 2021 and contributed significantly to parasitism. *Temelucha* sp., *C. ruficrus*, and *C. chlorideae*, on the other hand, were the least abundant parasitoids recovered from field-collected larvae throughout the study period. *C. formosanus* (12.55%) was the most active parasitoid and contributed to the highest for the total parasitism, followed by *Chelonus* nr. *blackburni* (10.98%). Surprisingly, nearly 2% of the field-collected larvae died due to entomopathogens infection. Furthermore, dead parasitoids accounted for 5.73 percent of total parasitism.

**Figure 3:**
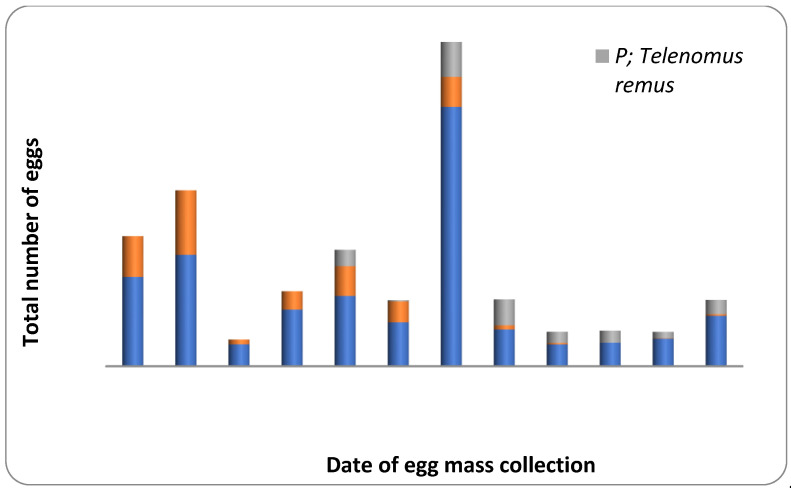
Parasitism level on Fall Armyworm eggs collected from maize agro-ecosystem during July–September, 2021. The orange color: Trichogramma chilonis; The grey color: Telenomus remus; The blue color: Unparasitized.

About 36.58 percent of the egg masses collected in the field was parasitized by egg parasitoids like *T. chilonis* and *T. remus* (Table 2; Figure 3). Natural parasitism caused by *T. chilonis* was 56.25 percent in July, 2021 and 24.05 percent in August, 2021. In comparison, the activity of *T. remus* was not recorded during July and August, 2021. However, the percentage natural parasitism due to *T. remus* was 23.48 during September, 2021. However, the percent of egg masses parasitized due to both the parasitoid was 7.41 and 6.96 during August and September, 2021, respectively. The activity of *T. chilonis* was observed during the third week of July 2021, and the incidence fluctuated throughout the crop growth period (Figure 3). In contrast, the incidence of *T. remus* started during the second week of August and remained active until the end of the crop growth period (Figure 3).

### 2.2. Effect of Different Botanicals on Developmental Stages of FAW

Significant differences were found among the botanicals with respect to its ovicidal (F_4,14_ = 26.183; *p* value < 0.0001) (Table 3), larvicidal, and pupicidal (Table 4) activity. The toxicity of botanicals at a uniform concentration was tested against FAW eggs, and the percent inhibition of egg hatching was recorded (Table 3). Eggs masses treated with double distilled water (control) hatched in 99 percent of the cases. All of the botanicals used in the study showed ovicidal activity for FAW eggs, but the percentage inhibition of hatching ranged from 4.69 to 100%. Essential oil of garlic was more toxic than other botanicals because, it completely inhibited hatching (100%) on FAW egg masses followed by geraniol (90.76 ± 8.88). Annona methanoic extract also recorded significant ovicidal action (60.63 ± 13.11). However, the geranium, picro, and Karanjin were least effective in inhibiting the hatching with 4.69 ± 5.15, 7.31 ± 3.34, and 8.35 ± 10.59 percentage, respectively.

Botanicals were toxic to *S. frugiperda* larvae at a single concentration (1%), and the maximum mortality was observed after 72 h. Geranium oil resulted in the highest mean larval mortality after 24 h of treatment (40%). Citronella oil treatment resulted in 60 and 90 percent mortality of treated larvae after 48 and 72 h, respectively. After 72 h of treatment, annona, geranium oil, geraniol, clove compound, sesame oil, chilli, garlic are equally effective (40%) against the *S. frugiperda*. Citronella oil and annona were most effective at achieving 100 percent mortality of exposed insect after 168 h, followed by citronellol and geranium oil (90%). However, annona methane extract and Annona acetone extract were found ineffective against larvae of *S. frugiperda*.

The essential oil of chilli caused significant larval mortality (40% at 168 h), but it led to phytotoxicity in exposed maize leaves. All of the survived larvae from each treatment were observed until pupation and adult emergence. All the survived larvae treated with citronellal and sesame oil went to pupation. The percentage of larvae that develop into pupae after being exposed to geraniol was the lowest (16.7). Aside from the toxicological effect, the botanicals of annona, citronellol, and clove had antifeedant activity on the treated larvae. Furthermore, adults emerged from sesame oil, and karanjin-treated larvae showed wing deformation. The adults emerged from the pupae treated with annona acetone extract (40%), and karanjin (30%) showed wing deformation.

### 2.3. Effect of Intercropping on the Incidence of FAW

The plants with excreta, damage severity scale, and numbers of live larvae per plant were recorded at 5, 6, 7, 8, and 9 weeks after sowing (WAS) and presented in Table 5. The percent plant damage of maize differed significantly between treatments at six (F_2,6_ = 3.830; *p* < 0.0023), seven (F_2,6_ = 7.307; *p* < 0.002), eight (F_2,6_ = 11.872; *p* < 0.0001), and nine weeks (F_2,6_ = 10.711; *p* < 0.0001). During 5 WAS, the percent incidence was non-significant in the intercropping systems. The percent plant incidence was highest in monocrop (33.02, 48.86, 61.99, and 67.10 percent) and lowest in maize + lady’s finger (11.39, 12.2, 12.74 and 13.81 percent), during 6, 7, 8, and 9 WAS, respectively (Table 6).

There was a significant difference in the number of live larvae between the intercropped and monocropped maize plots at 7 (F_2,6_ = 3.612; *p* < 0.0028), 8 (F_2,6_ = 10.231; *p* < 0.0001) and 9 WAS (F_2,6_ = 11.063; *p* < 0.0001). The least number of live larvae was recorded in maize + Lady’s finger intercropping in all the observed weeks. Monocrop of maize recorded the highest number of live larvae/plant *viz.,* 1.10, 1.01, and 1.28/plant during 7, 8, and 9 WAS, respectively. The severity of damage was non-significant among the different intercropping systems during 5 (F_2,6_ = 4.069; *p* < 0.019), and 6 WAS (F_2,6_ = 1.131; *p* < 0.401). Whereas, during 7 and 8WAS, the severity of damage was highest in control plots (5.95 ± 0.47 and 5.86 ± 0.39, respectively) but remained non-significant among the different intercropping systems.

**Figure 4:**
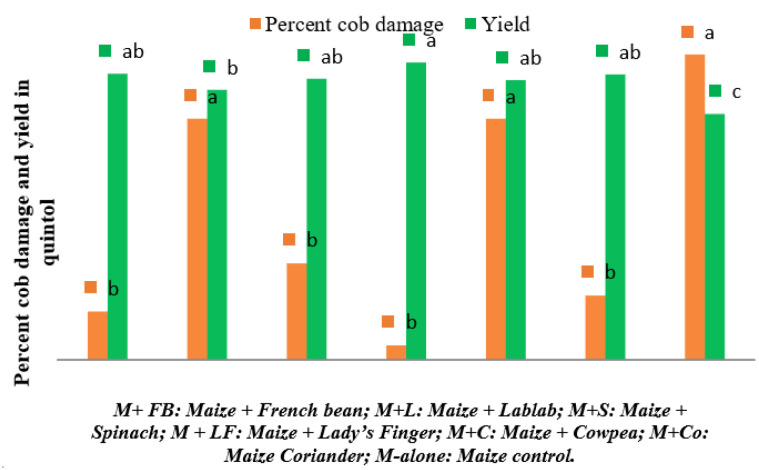
Percent cob damage by *S. frugiperda* and maize grain yield under different intercropping systems in New Delhi, India. [Bars of same colour marked with different letters differ significantly; *p* < 0.05, Duncan’s multiple range test].

Similarly, the highest number of plants carrying excreta was observed in control plots during 8 (29.00 ± 9.85) and 9 WAS (34.00 ± 16), however it remains non-significant among different intercropping systems during 9 WAS. Figure 4 depicts the percentage cob damage and yield under the intercropping systems. The cob damage was significantly superior in control plots (6.33) and lablab intercropped field (5.00), and the lowest cob damage was observed in the lady’s finger intercropping system (0.30). The maize + lady’s finger recorded highest grain yield (6.17 quintal/ha), whereas, the control plot recorded the lowest grain yield (5.13 quintal/ha).

### 2.4. Efficacy of Biopesticides against Larvae of FAW

**Figure 5:**
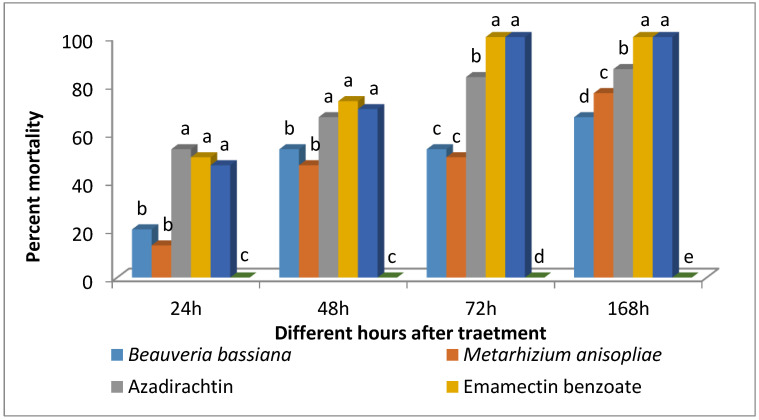
Efficacy of different biopesticides and insecticides against 3rd instar larvae of S. frugiperda. Bars marked with different letters differ significantly; *p* < 0.05, Duncan’s multiple range test.

The analysis of variance (ANOVA) revealed that the efficacy of biopesticides against *S. frugiperda* varied significantly after 24 h (F_2,9_ = 29.98; *p* < 0.0001), 48 h (F_2,9_ = 57.1; *p* < 0.0001), 72 h (F_2,9_ = 99.05; *p* < 0.0001), and 168 h (F_2,9_ = 252.6; *p* < 0.0001) of exposure to treatments (Figure 5). At 24 h after treatment, the mortality caused by Azadirachtin, Emamectin benzoate, and Chlorantraniliprole was equally significant. In contrast, mortality caused by *B. bassiana* and *M. anisopliae* was less effective, with no difference observed between them. A similar trend in mortality was observed at 48 h after treatment. All the larvae on Emamectin benzoate and Chlorantraniliprole treated leaves died at 72 h after treatment, while, Azadirachtin treated larvae showed 83.33% mortality. The overall efficacy of biopesticides formulation was in the following order azadirachtin > *M. anisopliae* > *B. bassiana* at 168 h after treatment.

## 3. Discussion

The FAW is a polyphagous exotic and invasive pest which causes significant crop damage and threatening food and nutritional security [5]. The sole reliance on chemical control not only eliminated the natural defender population, but also, led to a slew of environmental issues such as pesticide residues, resistance to pesticides, pest resurgence, and effects on non-target organisms [31]. The current study focuses on potential biological and maize agroecosystem manipulation (intercropping different plants) interventions for *S. frugiperda* management in India.

Eight different parasitoid species attacking the eggs and larvae of *S. frugiperda* were found in northern India. In similar surveys carried out in central and North India, Keerthi et al. [3] and Sagar et al. [20] documented the emergence of several parasitoids from central and northern India, respectively. Furthermore, Sharanabasappa et al. [18] reported five parasitoids from South India. Our results showed that larval parasitism levels ranged from 28.37 to 42.44% during the different months of larval collection. At the same time, the percent egg parasitism varied from 30.44 to 56.25. Similarly, Navik et al. [22] reported the parasitism of FAW eggs between 15.81–23.87 and 5.44–8.78 percent due to *T. chilonis* and *T. remus*, respectively.

Among the parasitoids reported in the present study, *C. formosanus* was the most dominant parasitoids (12.55) collected during the study period, followed by *Chelonus* nr. *blackburni* (10.98) and *Coccygydium* sp. (4.85). The members of *Chelonus* sp. are arrhenotokous, solitary, egg-larval parasitoids of several important lepidopterous pests distributed throughout the world. *C.* nr *blackburni* and *C*. *formosanus* are the widely distributed parasitoids of lepidopteran pests in the Neotropical and Oriental region [32]. However, Sagar et al. [20] reported *Chelonus* nr. *blackburni* was the most abundant parasitoid in northern India; higher sampling size carrying out in the present study might have contributed to the variations. Further, *Coccygidium* sp.(Hymenoptera: Braconidae) was one of the most dominating parasitoids identified in the study. The parasitoid was less active in the early cropping season, but it replaced other parasitoids and remained the most abundant in the later cropping season. *Coccygidium* sp. is known to parasitize the larvae of many noctuids, including *Spodoptera* spp. [33]. Many researchers have reported that, different species of *Coccygydium* sp. parasitized the larvae of *S. frugiperda* [33,34]. Average parasitism across India was higher than the parasitism rate reported in other studies of Fall Armyworm in corn (0.76%, Sagar et al. [20]; 0.001%, Sharanabasappa et al. [18]). However, the parasitism rate in the present study was lesser than what reported in the African countries (3.5–19.3, Agboyi et al. [35]; 9.2%, Otim et al. [36]). The variation in the parasitism might be due to the variable distribution of different species of *Coccygidium* sp. and also the different general equilibrium levels of FAW.

Two major egg parasitoids (*T. chilonis* and *T. remus*) have emerged from the field-collected egg masses. *T. chilonis* was the most dominant parasitoid reported in the early crop period, later replaced by *T. remus*. The *Trichogramma* sp. and *T. remus* are the most important parasitoid used in the biological control of *S. frugiperda* in Latin American countries such as Venezuela, Colombia, Brazil and Mexico [37]. The earlier workers in India also reported the field parasitization by the aforementioned parasitoids [21,22]. Competition is a dynamic, ongoing process. As a result, an interspecific competition that results in displacement has far-reaching evolutionary repercussions for the interacting species. Two species can survive for an extended period, but eventually, one will evolve better competitive powers, displacing the other [38,39,40]. The displacement might be due to variation in the temporal distribution of parasitoids and also the effect of environmental factors [41].

The plant botanicals are among the promising alternatives to synthetic pesticide as it offered added advantages like repellent, and antifeedant and that’s why, emphasized as an important tool in Integrated Pest Management (IPM) [28]. In the present work, we evaluated fourteen different botanicals against eggs, larvae, and pupae of *S. frugiperda.* The obtained results using different botanicals substantiate the literature and provide additional information on the biocidal potential of extracts and botanicals [28,42].

The botanicals like citronella oil and annona extracts were most effective, resulting in 100% mortality of *S. frugiperda* larvae. When botanicals having insecticidal property interact with insect integument, they may influence digestive and neurological enzymes [43]. The essential oil of the garlic was highly effective in inhibiting egg hatching (100%), followed by geraniol (90.76%). Allyl disulfide is an important component of the garlic oil and it is known for exhibiting ovicidal and larvicidal activity and the same might have contributed to ovicidal action against the eggs of *S. frugiperda* [44]. Similarly, geraniol also known to exhibit ovicidal action against the housefly eggs, which reduced the 99% of hatching [45]. Among the screened botanicals, none of the compounds are known to exhibit significant pupicidal activity against *S. frugiperda*. However, significant adult wing deformation was noticed in the pupae treated with annona acetone extract. The insecticidal activity of acetone extract of *Annona squamosa* L. (Magnoliales: Annonaceae) was also reported by several workers [46,47]. The differences in the insecticidal activity of different botanicals are primarily attributable to their composition and modes of action, which resulted in morphological, physiological, and behavioral changes in *S. frugiperda* at various growth stages [48,49].

In the present study, maize intercropped with lady’s finger recorded significantly the lowest percent plant damage during all the observed weeks. At the same time, the percent damage in maize intercropped with French bean, lablab, spinach, cowpea, and coriander was on par with each other. The present study was the first of its kind in evaluating the effect of vegetable intercropping on the incidence of FAW in India. However, the results of French bean and cowpea intercropping systems are in accordance with the findings of Hailu et al. [50] and Udayakumar et al. [21]. Because of the presence of natural enemies, maize intercropped with beans recorded a lower incidence [21]. The number of plants with excreta was significantly lower in the lady’s finger intercropped plot and highest in the control plot. The suppression of herbivore populations and reduction of damage has already been demonstrated in several intercropping systems; Saminathan et al. [51] documented the lowest pest activity and higher activity of natural defender population in cotton intercropped with lady’s finger. It is well known that insect pest populations are fewer in diverse ecosystems or intercrops [52,53]. The current study supports the previously reported findings that intercropped maize with leguminous and other crops resulted in much decreased FAW infestation compared to mono-cropped maize [50].

The infestation of FAW on maize cob was highest in the lablab and cowpea intercrops, and it is on par with the control plots. The percent cob damage was lowest in French bean, spinach, lady’s finger, and coriander intercropping system and on par with each other. Similarly, the highest grain yield was obtained in maize + lady’s finger intercropping system followed by French bean, spinach, cowpea, and coriander intercropping system and was on par with each other. Similar results were reported by Tanyi et al. [54], who stated the intercropping of maize with different beans reduced the incidence of *S. frugiperda*. Pest management through habitat manipulation via intercropping system was established in many cropping systems. It was based on the hypothesis that confusing olfactory and visual cues received from the companion beans plants probably served as the push component that repelled FAW larvae away from the maize plants. In the case of the lady’s finger plot, the green leaf volatiles emitted by it might be repelling the FAW adults and larvae to move away from maize [54]. The low maize yield recorded in control plots was consistent with the FAW severity and maize infestation. In contrast, the highest grain yield was recorded in the maize + lady’s finger intercropping system. The improved maize yield in the plots intercropped with beans could be due to a combination of low FAW severity and maize infestation [54]. The disparity observed is probably due to the crop morphology, ecosystem, and management practices [53].

In laboratory bioassays, moderate to high larval mortality was achieved with *M. anisopliae*, *B. bassiana,* Azadirachtin, Emamectin benzoate, and Chlorantraniliprole. Bio-pesticides are vital in IPM and are the best alternative to chemical control [31]. Azadirachtin was the most effective against the larvae among the biopesticides, followed by *M. anisopliae* and *B. bassiana.* The results follow the earlier findings of Dhobi et al. [55]. Synthetic insecticides are important management options in FAW control, as is common with other insect pest species. In India, chemical control of FAW in maize is achieved by applying Cyantraniliprole, Emamectin benzoate, and Thiamethoxam, among other synthetic insecticides [8]. Emamectin benzoate and Chlorantraniliprole are highly effective against the *S. frugiperda* resulting in 100% mortality after 72 h after exposure. Deshmukh et al. [56] also evaluated the field efficacy of insecticides and reported chlorantraniliprole was the most effective, followed by emamectin benzoate.

## 4. Materials and Methods

### 4.1. Insect Rearing

The rearing was established from larvae collected in experimental maize fields of ICAR- Indian Agricultural Research Institute, Pusa, New Delhi, and maintained using an artificial diet proposed by Gujar et al. [57] with few modifications (Chickpea flour was reduced to 90 g instead of 108 g, and streptomycin sulphate was increased to 0.48 g instead of 0.2 g). The field collected larvae were reared for two generations under laboratory conditions, and the successive developmental stages of FAW were used for laboratory evaluations.

### 4.2. Field Collection of FAW (Egg Masses and Larvae) and Laboratory Rearing

Egg masses and larvae of FAW were collected from different maize fields of ICAR- Indian Agricultural Research Institute (IARI), Pusa, New Delhi (28.08° N, 77.12° E) during July–October 2021. Plants with visible FAW attack were selected in each field and checked for the presence of FAW egg masses and larvae. Larvae were pulled from the whorl and placed individually in small Petri dishes (2 cm height × 5.8 cm diameter) containing an artificial diet until parasitoid emergence. To avoid damage to any eggs in the batch, the FAW egg masses were collected from infested maize plants along with a piece of fresh leaves. The individual egg mass was placed in Petri dishes. The collected egg masses and larvae were maintained in the insect rearing laboratory under climatized room (27 ± 1 °C, 65 ± 5% RH and 14 h: 10 h L: D photophase), in ICAR-IARI, New Delhi. The parasitoids that emerged from the collected larvae were regularly preserved in 70% alcohol. At every week, an attempt was made to collect at least 250 larvae and 25 egg masses; however, larger or smaller numbers of larvae and egg masses were collected during some weeks.

### 4.3. Evaluation of Botanicals against FAW

Bioassays were carried out to evaluate the activity of 14 different botanicals on the egg (*n* = 5 egg masses per treatment), larvae (*n* = 10), and pupae (*n* = 10) of FAW in a Completely Randomized Design (CRD) under laboratory conditions. Each treatment of botanicals on different developmental stages of FAW was replicated thrice. The negative control consisted of distilled water, and 5.0% (*v*/*v*) of neutral detergent (Tween 80) used to correct the results obtained with botanicals.

#### 4.3.1. Preparation of Plant Extracts

The mature and dried seeds of custard apple, *Annona squamosa* L. and sesame, *Sesamum indicum* L. (Lamiales: Pedaliaceae) were purchased from the local market, cleaned and powdered. The powdered seeds of *A. squamosa* were extracted sequentially with hexane, acetone and methanol to obtained different extracts while sesame oil was obtained from the powdered sesame seeds with hexane using Soxhlet apparatus. The solvents from the different extracts were removed using rotary evaporator to obtain the dried extracts.

#### 4.3.2. Chemicals and Solvent

Geranium oil and citronella oil (*Cymbopogon* spp.: Poales: Poaceae) were purchase from M/s Shiv Sales Corporation, New Delhi, citronellol and citronellal (*Cymbopogon* spp) from M/s SRL Pvt. Ltd., India, while tween-80 from M/s Ranchem Pvt Ltd., India. Garlic oil (*Allium sativum* L. Plantae: Amaryllidaceae) and *Capsicum* oleoresin (*Capsicum* spp. Solanales: Solanaceae) were obtained from M/S Bio-India Biologicals, Corporation, Hyderabad. Geraniol, oleanolic acid and karanjin were isolated and identified from palmarosa oil (*Cymbopogon martini* (Roxb.): Poales: Poaceae), clove buds (*Syzygium aromatic* (L.) Merr. & L.M.: Myrtales: Myrtaceae) and karanj seed (*Millettia pinnata* (L.) Panigrahi, Fabales: Fabaceae).

#### 4.3.3. Preparation of Test Solution

Stock solution (1.0%) of the oils, pure compounds and extracts were prepared with Tween-80 (3.0%) in distilled water using lab stirrer by stirring the content for 1 h. A uniform concentration of 1% was used for all the botanicals.

#### 4.3.4. Bioassay for Ovicidal Activity

The eggs of same age groups (*n* = 5) were collected from the laboratory culture, and each egg mass was sprayed with different botanicals (1%) using hand held atomizer. Only a desired quantity (2 mLfor each egg mass) of botanicals was used for uniform application and sprayed on the FAW egg masses. After spraying, the egg mass was individually transferred into Petri dishes. The deterioration or hatching of *S. frugiperda* eggs was observed after 7 days of treatment by counting the dead larvae and unhatched eggs in each Petri plate. The hatchability percent was worked out as per the following formula as given by Sangha et al. [58].

(1) $$\mathrm{Hatchability}\;\mathrm{percentage}=\frac{\mathrm{Number}\;\mathrm{of}\;\mathrm{dead}\;\mathrm{larvae}\;\mathrm{in}\;\mathrm{each}\;\mathrm{petriplate}}{\mathrm{Unhatched}\;\mathrm{eggs}+\mathrm{Number}\;\mathrm{of}\;\mathrm{dead}\;\mathrm{larvae}}$$

#### 4.3.5. Larvicidal Activity Bioassay by Leaf Dipping Method

Under this, 1% botanicals solution was taken in the Petri dish (9 cm). The leaf disc of maize (5 cm diameter) was dipped in the solution for a minute and later shade dried before exposing larvae for feeding. Accumulated mortalities were assessed at 24, 48, 72, and 168 h after application of treatments including control. Observed mortality was also corrected for the control using the formulas proposed by Abbott [59]. After 72 h, the surviving larvae in each treatment were transferred to new Petri dishes containing an artificial diet. Observations were made on the remaining larvae to document the number of pupae formed (larval viability) and the number of adults formed (pupal viability). During the observations, the presence of feeding marks on the leaves and fecal pellets in the Petri dishes was noted for the antifeedant activity. In addition, the phytotoxicity effect of botanicals on the maize leaves was observed by recording the change of leaf colour, leaf drying, and early loss of turgidity by comparing it with the control.

#### 4.3.6. Bioassay for Pupicidal Activity

Pupicidal activity (Baskar et al. [60]) was determined using pupae (48 h old) of *S. frugiperda*. Pupae (batch of 10) were placed on filter paper and sprayed with 2 mLof different botanicals solutions and control using an atomizer. After 30 min, the pupae were transferred to Petri dishes (9 cm diameter) lined with filter paper. The observations on percent adult emergence and the wings deformations among the emerged adults were recorded.

### 4.4. Effect of Intercropping on the Incidence and Damage by FAW

**Figure 6:**
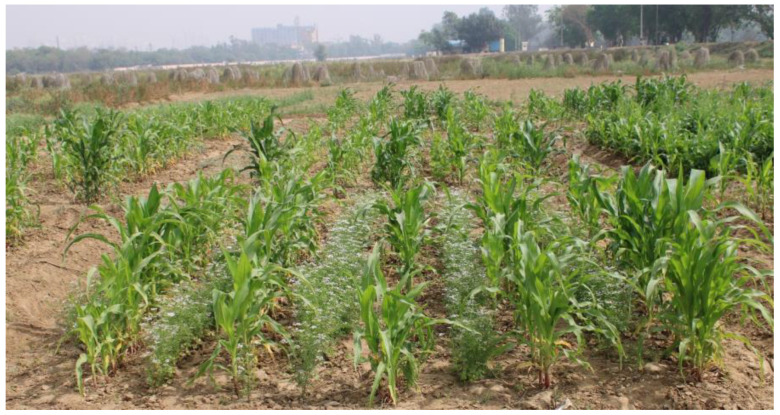
Maize + coriander, Coriander sativum intercropping system.

**Figure 7:**
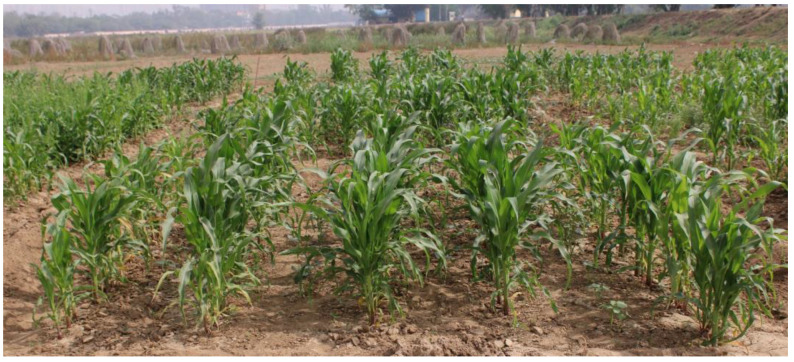
Maize + Lady’s finger, *Abelmoschus esculentus* intercropping system.

The experiment was conducted in the winter season of 2020 in Randomized Block Design (RBD) with a plot size of 5 m × 5 m. The treatments were seven and replicated thrice. The different treatments were maize + coriander (*Coriander sativum* L.; Figure 6), maize + lablab (*Lablab purpureus* (L.) Sweet), maize + French bean (*Phaseolus vulgaris* L.), maize + vegetable cowpea (*Vigna unguiculata* (L.) Walp.), maize + lady’s finger (*Abelmoschus esculentus* L.; Figure 7), maize + spinach (*Spinacia oleracea* L.) and maize as solo crop. The maize crop was planted with a 75 × 20 cm inter-row and intra-row spacing.

Intercrops were sown in between the rows of maize in the intercropped plot. The total plants in each plot were observed for the damage signs during each week. The percent plant damage was calculated by dividing the total number of plants in the plot by the number of damaged plants. The leaf damage caused by *S. frugiperda* was assessed visually and scored on a 0–9 scale [61]. In addition, total numbers of plants having excreta were also recorded during each observed week. In the later growth stage, the total numbers of cobs damaged in each plot were noted. The yield of cobs in each plot was recorded, and the yield/acre was calculated by extrapolation.

### 4.5. Evaluation of Selected Bio-Pesticides against FAW

The current study was conducted in the biocontrol laboratory, Division of Entomology, ICAR-IARI, Pusa, New Delhi, in *Kharif*, 2021. Treatments comprised of three biopesticides *viz*., *Beauveria bassiana* (Balsamo) Vuillemin1.15% WP (1×10^8^ CFU/g at 5g/liter) (Hypocreales: Clavicipitaceae), *Metarhizium anisopliae* (Mechnikov) Sorokin 1% WP (Balsamo) Vuillemin (Hypocreales: Clavicipitaceae) (1×10^8^ CFU/g at 5g/liter) and Azadirachtin E.C. 1% *w*/*w* (10,000 ppm) at 1mL/liter. In addition, two insecticides *viz*., Emamectin benzoate 5SG and Chlorantraniliprole 18.5SC were used as a positive control and water as a negative control. The leaf dip (5 cm diameter) bioassay method was followed to evaluate the toxicity against 3rd instar larvae of *S. frugiperda*. The fresh leaves of maize were made into leaf discs then dipped in the different insecticides and shade dried; later, were offered to the 12 h starved larvae of *S. frugiperda* (*n* = 10). The post treatment observations on surviving larvae were recorded at 24, 48, 72, and 168 h after treatment. The observations recorded were analyzed statistically using Abbott’s formula [59] to determine the corrected mortality and the relative efficacy of different biopesticides used in the study was analyzed.

### 4.6. Data Analysis

Analysis of variance-one way [62] was used to compare the effect of intercrops on the percent pest incidence, the number of live larvae per damaged plants, plants showing excreta, where significant difference was detected treatment means were separated using Duncan’s multiple range test (0.5%). The percent pest incidence values are arc signed, while live larvae per plant and plant with excreta are square transformed.

Larval mortality due to unknown factors, microbial infections, adult emergence and larval parasitisation rates were calculated for all observed months. The formula [63], $PR=\frac{Pi}{Pt}$ was used to calculate the parasitism rate of parasitoids, where *P_i_* is the number of parasitized individuals of species *i* and *P_t_* the total number of larvae collected. Similarly, the formula [64], $RA=\frac{Ni}{Nt}$ was used to calculate the relative abundance of a particular parasitoid (*RA*), where *Ni* is the number of individuals of each parasitoid species and *N_t_* is the total number of parasitoids emerged from field collected larvae. The relative contribution of each parasitoid species to total parasitism (*RP*) was calculated by using $=\frac{PS}{LS}$; Where, *PS* = total number of FAW larvae parasitized by each parasitoid; *LS* = total number of FAW larvae collected.

## 5. Conclusions

The incidence of Fall Armyworm ranged from 33.02 to 67.10% and the total parasitism rate due to all parasitoids ranged from 28.37 to 42.44%, indicating the potential for biological control through conservation of natural enemies. In the study area, eight different parasitoid species attacked the eggs and larvae of *S. frugiperda*. Among these parasitoids, *C. formosanus* and *C. blackburni* were the most important egg larval parasitoids, and *T. remus* and *T. chilonis* were the most important egg parasitoids. Under laboratory conditions, essential oil of citronella and annona extract were effective against *S. frugiperda* larvae, while essential oil of garlic showed strong ovicidal action against *S. frugiperda*. However, field efficacy must be validated. The maize + lady’s finger intercropping system had the lowest infestation and the highest grain yield. Among the commercial biopesticides evaluated, azadirachtin 10,000 ppm was effective against larvae of *S. frugiperda*, followed by *Metarhizium anisopliae*.

## Figures and Tables

**Table 1:** Relative abundance and parasitism rate of native parasitoids on *S. frugiperda* in New Delhi, India.

Parasitoid Species	Host Stage Attacked	July, 2021 (*n* = 926)			August, 2021 (*n* = 1036)			September, 2021 (*n* = 557)			October, 2021 (*n* = 222)			Relative Contribution to Total Parasitism (RP)
		PE	RA (%)	PR (%)	PE	RA (%)	PR (%)	PE	RA (%)	PR (%)	PE	RA (%)	PR (%)	
*Chelonus* nr. *blackburni*	Egg-larva	175	43.77	18.57	125	32.30	12.07	4	2.53	0.72	0	-	-	10.98
*Chelonus formosanus*	Egg-larva	126	32.06	13.61	126	32.56	12.16	72	45.57	12.93	20	29.85	9.01	12.55
*Coccygidium* sp.	Larva	10	2.54	1.08	53	13.70	5.12	47	29.75	8.44	23	34.33	10.36	4.85
*Temelucha* sp.	Larva	12	3.05	1.30	42	10.85	4.05	5	3.16	0.90	0	-	-	2.15
*Cotesia ruficrus*	Larva	3	0.76	0.32	1	0.26	0.10	0	-	-	0	-	-	0.15
*Campoletis chlorideae*	Larva	3	0.76	0.32	3	0.78	0.29	1	0.63	0.18	0	-	-	0.26
Dead parasitoids from field collected larvae	Egg-larva/Larva	67	17.05	7.24	37	9.56	3.57	29	18.35	5.21	24	35.82	10.81	5.73
Larva infected with entomopathogens	Larva	11	-	-	32	-	-	9	-	-	-	-	-	1.90
Total dead larvae	Larva	59	-	-	122	-	-	108	-	-	42	-	-	
Total PR (%) (*n* = 2741)		42.44			37.36			28.37			30.18			

PE: Number of individual parasitoids emerged from the field collected larvae; RA: Relative abundance of parasitoids; PR: Percent parasitism. *n* = total number of larvae collected during each month.

**Table 2:** Percentage of Fall Armyworm egg mass parasitized by *Trichogramma chilonis* and *Telenomus remus*.

Parasitoids	July, 2021 (*n* = 64)	August, 2021 (*n* = 108)	September, 2021 (*n* = 115)	Total Egg Mass Collected (287)
	% Egg MassParasitized	% Egg Mass Parasitized	% Egg Mass Parasitized	% of Total Egg Mass Parasitized
*T. chilonis*	56.25	24.05	-	21.60
*T. remus*	-	-	23.48	5.57
*T. chilonis + T. remus*	-	7.41	6.96	9.41
Total percent parasitized	56.25	31.46	30.44	36.58

*n* = total number of egg masses collected during each month.

**Table 3:** Ovicidal action of different botanicals on eggs of Fall Armyworm.

Sl. No	Botanicals (1%)	% Egg Hatch	% Inhibition of Egg Hatch
1	Citronellal	65.41 ± 15.25 ^cd^	34.59 ± 15.25 ^cd^
2	Citronella Oil	64.03 ± 12.39 ^cd^	35.97 ± 12.39 ^cd^
3	Annona	88.61 ± 21.61 ^ab^	11.39 ± 21.61 ^ef^
4	Citronellol	87.60 ± 16.79 ^ab^	12.40 ± 16.79 ^ef^
5	Geranium Oil	95.31 ± 5.15 ^a^	4.69 ± 5.15 ^f^
6	Picro	92.69 ± 7.34 ^a^	7.31 ± 7.34 ^f^
7	Geraniol	9.24 ± 8.88 ^f^	90.76 ± 8.88 ^a^
8	Clove, *Syzygium aromaticum*	59.91 ± 19.53 ^cd^	40.09 ± 19.53 ^cd^
9	Annona methanoic Extract	39.37 ± 13.11 ^e^	60.63 ± 13.11 ^b^
10	Sesame Oil	87.13 ± 12.38 ^ab^	12.87 ± 12.38 ^ef^
11	Chilli	50 ± 23.59 ^de^	50 ± 23.59 ^bc^
12	Karanjin	91.65 ± 10.59 ^a^	8.35 ± 10.59 ^f^
13	Garlic	0.00 ^f^	100 ± 0.00 ^a^
14	Annona Acetone Extract	74.13 ± 8.80 ^bc^	25.87 ± 8.80 ^de^
15	Control	99.02 ± 1.53 ^a^	0.98 ± 1.53 ^f^
*F* value		26.183	26.183
*p* value		<0.0001	<0.0001

Values in table marked with different letters differ significantly; *p* < 0.05, Duncan’s multiple range test.

**Table 4:** Percent mortality of larvae and pupae of Fall Armyworm after exposure to different botanicals.

Sl. No	Botanicals (1%)	% Mortality of Fall Armyworm Larvae							% Mortality of Pupae	Other Remarks
		24 h	48 h	72 h	168 h	Larval Viability	Pupal Viability	Other Remarks		
1	Citronellal	0 ^a^	30 ^abc^	30 ^abc^	30 ^abc^	100	85.7	-	-	-
2	Citronella oil	10 ^a^	60 ^c^	90 ^d^	100 ^e^	0	0	-	10	-
3	Annona	0 ^a^	10 ^ab^	40 ^bc^	100 ^e^	0	0	AFA	-	-
4	Citronellol	10 ^a^	10 ^ab^	20 ^abc^	90 ^de^	100	100	AFA	-	-
5	Geranium oil	40 ^b^	40 ^bc^	40 ^bc^	90 ^de^	100	100	-	10	-
6	Picro	10 ^a^	10 ^ab^	10 ^ab^	10 ^ab^	88.9	62.5	-	-	AWD (20%)
7	Geraniol	10 ^a^	20 ^ab^	40 ^bc^	40 ^bc^	16.7	100	-	-	AWD (10%)
8	Clove	0 ^a^	20 ^ab^	40 ^bc^	50 ^c^	60.0	66.7	AFA	-	EM
9	Annona methane extract	0 ^a^	10 ^ab^	10 ^ab^	10 ^ab^	77.8	71.4	-	-	-
10	Sesame oil	10 ^a^	10 ^ab^	40 ^c^	50 ^c^	100	60	AWD	10	AWD (20%)
11	Chilli	0 ^a^	0 ^a^	40 ^bc^	40 ^bc^	66.7	50	PTE	-	EM
12	Karanjin	0 ^a^	0 ^a^	10 ^ab^	10 ^ab^	100	88.9	AWD	10	AWD (30%)
13	Garlic	10 ^a^	20 ^ab^	40 ^bc^	60 ^cd^	75.0	66.7	-	-	-
14	Annona acetone extract	0 ^a^	0 ^a^	10 ^ab^	10 ^ab^	77.8	85.7	-	-	AWD (40%)
15	Control	0 ^a^	0 ^a^	0 ^a^	0 ^a^	100	100	-	-	-
	CV	24.05	41.23	62.7	70.7					
	CD @5% and @1%	0.198 * NS@1%	0.304 *; 0.399 **	0.378 *; 0.497 **	0.335 *; 0.440 **	-	-	-	-	-

AFA: Antifeedant activity observed; AWD: Adult wing deformation observed; PTE: Severe phytotoxicity effect on maize leaf; EM: Early mortality of adults. * CD @5% and ** CD @1%; NS: Values in table marked with different letters differ significantly; *p* < 0.05, Duncan’s multiple range test; Non-significant.

**Table 5:** Mean number of larvae, damage scale and mean number of plants with excreta in intercropped and sole crop of maize.

Treatments	5WAS		6WAS			7WAS			8WAS			9WAS		
	NOL	DS	NOL	DS	PWE	NOL	DS	PWE	NOL	DS	PWE	NOL	DS	PWE
M + FB	0.22 ± 0.38	0.78 ± 1.35 ^b^	0.83 ± 0.17	2.83 ± 2.52	0.67 ± 1.15	0.78 ± 0.05 ^a^	3.96 ± 1.03 ^b^	11.33 ± 1.53 ^b^	0.72 ± 0.04 ^b^	3.61 ± 0.53 ^b^	19.00 ± 9.64 ^ab^	0.54 ± 0.13 ^b^	3.65 ± 0.33 ^b^	8.33 ± 2.08 ^b^
M + L	0.00	0.00 ^b^	0.74 ± 0.22	2.55 ± 2.36	0.00	0.70 ± 0.17 ^a^	3.95 ± 0.55 ^b^	13.00 ± 2 ^b^	0.56 ± 0.09 ^c^	3.85 ± 0.69 ^b^	15.33 ± 4.04 ^bc^	0.64 ± 0.01 ^b^	3.46 ± 0.77 ^b^	12.33 ± 4.04 ^b^
M + S	0.22 ± 0.38	1.11 ± 1.92 ^b^	0.90 ± 0.09	4.22 ± 0.28	1.00 ± 1.73	0.84 ± 0.01 ^a^	3.44 ± 0.76 ^b^	12.00 ± 2.65 ^b^	0.69 ± 0.06 ^bc^	3.85 ± 0.79 ^b^	9.33 ± 6.66 ^bc^	0.68 ± 0.06 ^b^	3.82 ± 0.28 ^b^	8.00 ± 2.65 ^b^
M + LF	0.33 ± 0.58	0.00 ^b^	0.53 ± 0.47	2.05 ± 1.85	0.00	0.70 ± 0.17 ^a^	2.96 ± 0.14 ^b^	1.67 ± 0.58 ^c^	0.57 ± 0.14 ^c^	3.29 ± 1.21 ^b^	5.33 ± 5.13 ^c^	0.61 ± 0.1 ^b^	3.51 ± 0.08 ^b^	6.67 ± 1.15 ^b^
M + C	0.27 ± 0.46	1.00 ± 1.73 ^b^	0.82 ± 0.06	4.18 ± 0.55	1.67 ± 1.53	0.85 ± 0 ^a^	4.06 ± 0.55 ^b^	17.33 ± 7.57 ^ab^	0.70 ± 0.1 ^bc^	3.81 ± 1.29 ^b^	18.67 ± 7.23 ^ab^	0.70 ± 0.09 ^b^	4.49 ± 0.88 ^ab^	7.33 ± 4.04 ^b^
M + Co	0.00	0.00 ^b^	0.88 ± 0.1	3.95 ± 0.65	1.00 ± 1	0.67 ± 0.20 ^a^	3.51 ± 1.20 ^b^	11.00 ± 1 ^b^	0.66 ± 0.01 ^bc^	3.06 ± 0.06 ^b^	11.00 ± 5.29 ^bc^	0.68 ± 0.16 ^b^	3.37 ± 0.25 ^b^	8.67 ± 1.53 ^b^
M-alone	0.49 ± 0.43	4.07 ± 1.01 ^a^	0.94 ± 0.05	3.57 ± 1.22	2.33 ± 2.08	1.10 ± 0.19 ^b^	5.95 ± 0.47 ^a^	22.67 ± 10.79 ^a^	1.01 ± 0.08 ^a^	5.86 ± 0.39 ^a^	29.00 ± 9.85 ^a^	1.28 ± 0.26 ^a^	5.71 ± 1.12 ^a^	34.00 ± 16 ^a^
*p* value	0.768 (NS)	0.019	0.420 (NS)	0.401 (NS)	0.356 (NS)	0.028	0.016	0.007	0.0001	0.035	0.026	0.0001	0.012	0.003
*F* value	0.540	4.069	1.092	1.131	1.231	3.612	4.234	5.283	10.231	3.378	3.701	11.063	4.543	6.387

M + FB: Maize + French bean; M + L: Maize + Lablab; M + S: Maize + Spinach; M + LF: Maize + Lady’s Finger; M + C: Maize + Cowpea; M + Co: Maize Coriander; M-alone: Maize control. WAS: Week after sowing; NOL: Number of larvae/plant; DS: Mean damage scale; PWE: Plants with excreta; Values in table marked with different letters differ significantly; *p*< 0.05, Duncan’s multiple range test; NS—Non-significant.

**Table 6:** Effect of vegetable intercropping on maize infestation by Fall Armyworm at different weeks after sowing (WAS).

	5WAS	6WAS	7WAS	8WAS	9WAS
Maize + French bean	2.63	12.29 ^bc^	18.57 ^bc^	27.07 ^b^	32.24 ^b^
Maize + Lablab	0.00	15.98 ^bc^	25.61 ^b^	32.47 ^b^	32.07 ^b^
Maize + Spinach	1.33	18.61 ^bc^	22.57 ^bc^	27.80 ^b^	30.62 ^b^
Maize + Ladies Finger	1.28	11.39 ^c^	12.25 ^c^	12.74 ^c^	13.81 ^c^
Maize + Cowpea	2.31	18.15 ^bc^	30.52 ^b^	35.04 ^b^	30.01 ^b^
Maize + Coriander	0.00	23.34 ^ab^	27.86 ^b^	30.33 ^b^	30.82 ^b^
Monocrop of maize	2.52	33.02 ^a^	48.86 ^a^	61.99 ^a^	67.10 ^a^
*F* value	0.781 (NS)	3.830	7.307	11.872	10.711
*p* value	0.601	*p <* 0.023	*p <* 0.002	*p <* 0.0001	*p <* 0.0001

WAS: Weeks after sowing. Values in table marked with different letters differ significantly; *p* < 0.05, Duncan’s multiple range tests. NS—Non-significant

## Data Availability

All data included in the main text.
